# Multisystem proteinopathies (MSPs) and MSP‐like disorders: Clinical‐pathological‐molecular spectrum

**DOI:** 10.1002/acn3.51751

**Published:** 2023-03-01

**Authors:** Pitcha Chompoopong, Björn Oskarsson, Nicolas N. Madigan, Igal Mirman, Jennifer M. Martinez‐Thompson, Teerin Liewluck, Margherita Milone

**Affiliations:** ^1^ Department of Neurology Mayo Clinic Rochester Minnesota USA; ^2^ Department of Neurology Mayo Clinic Jacksonville Florida USA

## Abstract

**Objectives:**

Mutations in *VCP, HNRNPA2B1, HNRNPA1*, and *SQSTM1*, encoding RNA‐binding proteins or proteins in quality‐control pathways, cause multisystem proteinopathies (MSP). They share pathological findings of protein aggregation and clinical combinations of inclusion body myopathy (IBM), neurodegeneration [motor neuron disorder (MND)/frontotemporal dementia (FTD)], and Paget disease of bone (PDB). Subsequently, additional genes were linked to similar but not full clinical‐pathological spectrum (MSP‐like disorders). We aimed to define the phenotypic‐genotypic spectrum of MSP and MSP‐like disorders at our institution, including long‐term follow‐up features.

**Methods:**

We searched the Mayo Clinic database (January 2010–June 2022) to identify patients with mutations in MSP and MSP‐like disorders causative genes. Medical records were reviewed.

**Results:**

Thirty‐one individuals (27 families) had pathogenic mutations in: *VCP* (*n* = 17), *SQSTM1 + TIA1* (*n* = 5), *TIA1* (*n* = 5), *MATR3, HNRNPA1, HSPB8*, and *TFG* (*n* = 1, each). Myopathy occurred in all but 2 *VCP*‐MSP patients with disease onset at age 52 (median). Weakness pattern was limb‐girdle in 12/15 *VCP*‐MSP and *HSPB8* patient, and distal‐predominant in other MSP and MSP‐like disorders. Twenty/24 muscle biopsies showed rimmed vacuolar myopathy. MND and FTD occurred in 5 (4 *VCP*, 1 *TFG*) and 4 (3 *VCP*, *1 SQSTM1 + TIA1*) patients, respectively. PDB manifested in 4 *VCP*‐MSP. Diastolic dysfunction occurred in 2 *VCP*‐MSP. After 11.5 years (median) from symptom onset, 15 patients ambulated without gait‐aids; loss of ambulation (*n* = 5) and death (*n* = 3) were recorded only in *VCP*‐MSP.

**Interpretation:**

*VCP*‐MSP was the most common disorder; rimmed vacuolar myopathy was the most frequent manifestation; distal‐predominant weakness occurred frequently in non‐*VCP*‐MSP; and cardiac involvement was observed only in *VCP*‐MSP.

## Introduction

Multisystem proteinopathies (MSPs) are rare genetically heterogenous disorders manifesting with inclusion body myopathy (IBM), neurodegeneration [amyotrophic lateral sclerosis (ALS)/ frontotemporal dementia (FTD)], and Paget disease of bone (PDB),^1^, ^2^ co‐existing in the same individual or co‐segregating in the same family.^3^, ^4^, ^5^ Despite the genetic heterogeneity, MSPs share underlying cellular defects which lead to abnormal cytoplasmic protein aggregation in affected tissues.^3^, ^6^, ^7^ Such protein aggregates display ubiquitin‐positive inclusions containing RNA‐binding proteins, such as TAR DNA binding protein‐43 (TDP‐43), and also ubiquitin‐dependent autophagy proteins, such as p62.^2^, ^8^

*VCP, HNRNPA2B1, HNRNPA1*, and *SQSTM1*, were the first set of genes recognized as causative of MSPs and classified as MSP1, MSP2, MSP3, and MSP4, respectively.^2^, ^3^, ^4^, ^9^ These genes encode RNA‐binding proteins or proteins with crucial role in quality‐control pathways. Subsequently, additional genes were linked to a similar clinical‐pathological spectrum featuring combination of at least 2 of 4 conditions (IBM, ALS, FTD, PDB), not necessarily in the same family. These genes were proposed to cause MSP subtypes and include: *MATR3* (distal myopathy with vocal cord paralysis and ALS),^10^, ^11^ *TIA1* (Welander distal myopathy and ALS/FTD),^1^, ^12^, ^13^ and *ANXA11* (IBM and ALS/FTD).^14^ These three genes have not been associated with PDB; isolated *TIA1* mutations (without co‐existing pathogenic *SQSTM1* variant) have not been reported as causative of both myopathy and ALS/FTD within the same pedigree. While MATR3 and TIA1 are RNA‐binding proteins,^10^, ^12^ ANXA11 is a calcium‐dependent phospholipid‐binding protein, not falling within the functional groups of other proteins associated with MSP. *OPTN*, encoding a protein involved in ubiquitin‐dependent autophagy, may also represent another MSP gene as *OPTN* mutations were reported as causative of ALS and risk factor for PDB, but not as causative of myopathy.^15^, ^16^ Additionally, *HSPB8* and *TFG* were associated with motor neuronopathy, sensory neuropathy, rimmed vacuoles on muscle biopsy, and could represent MSP genes. While description of TFG‐related muscle pathology is limited (rimmed vacuoles in 1 patient^17^ and sarcoplasmic TDP43/p62‐positive inclusions in 2 patients^18^), myopathological features of *HSPB8*‐related disorder are well documented.^19^, ^20^, ^21^, ^22^ TFG chaperones misfolded proteins into the ubiquitin‐proteasome system and its most common mutation (p.Pro285L) damages the protein quality control^23^; HSPB8 is also a chaperone participating in maintaining integrity and dynamics of stress granules,^24^ functions shared with other MSP causing proteins. Lastly, there are genetic disorders manifesting only with neurodegeneration, such as *TARDPB*‐related ALS/FTD, also featuring TDP‐43 mislocalization and ubiquitinated inclusions,^25^ but not classified as MSPs due to lack of other system (muscle and bone) involvement.

The rarity of MSPs, complex phenotypic spectrum including intrafamilial and inter‐familial variability, and genetic heterogeneity make their diagnosis challenging. Limited data are available on disease natural history and mostly from *VCP*‐MSP.

This study aims to describe the spectrum of MSPs and MSP‐like disorders with their clinical, pathological, and molecular correlates, as well as long‐term follow‐up, based on retrospective data review from time of diagnosis to last follow‐up at a single tertiary center.

## Methods

This study was approved by the Mayo Clinic Institutional Review Board (IRB 22–001886). We searched the Mayo Clinic data explorer from January 1, 2010, to June 30, 2022, to identify patients with mutations in genes known to cause MSPs (*VCP, HNRNPA2B1, HNRNPA1, SQSTM1*) and MSP‐like disorders (*MATR3, TIA1, ANXA11, OPTN, HSPB8*, and *TFG*). The search targeted only patients with genetically proven disease. Most patient underwent a next generation sequencing (NGS) panel targeting genes causative of neuromuscular diseases or ALS/FTD except for the single patient with *HSPB8* pathogenic mutation who underwent whole exome sequencing. Medical records were reviewed for demographic data, clinical presentation, laboratory, radiological, and histopathological findings. Race and ethnicity were self‐reported by patients. The diagnosis of myopathy was based on presence of objective muscle weakness by clinical examination, presence of myopathic changes on electromyography (EMG), and/or muscle biopsy findings. Myopathy with rimmed vacuoles was diagnosed when vacuoles rimmed by membranous or granular materials represented the predominant pathologic feature, at interpreting pathologist's discretion. To assess frequency of rimmed vacuoles, diagnostic slides were retrospectively reviewed to count and report number of vacuoles per 20X power field (mean ± SD). Areas of the sections mainly occupied by fibrous‐fatty connective tissue were excluded. The presence of angulated atrophic fibers of either histochemical fiber type overreactive for nonspecific esterase and target formations were interpreted as signs of denervation atrophy while fiber type grouping suggested reinnervation. A diagnosis of motor neuron disease (MND) was based on clinical evidence of upper motor neuron (hyperreflexia, Babinski sign, spasticity) or lower motor neuron (fasciculations, muscle weakness and atrophy) signs, and electrodiagnostic evidence of lower motor neuron involvement. Co‐existing neuropathy was noted if nerve conduction studies revealed reduced or absent sensory nerve action potentials or slow nerve conduction velocity in upper and lower limbs as well as length‐dependent sensory deficits on examination. Cognitive impairment was determined by bedside cognitive evaluation (Kokmen mental status short test) and formal neuropsychological testing. The diagnosis of FTD was based on neuropsychological findings of behavioral alteration (e.g., apathy, perseveration, and disinhibition), early expressive/receptive aphasia with relatively preserved memory, orientation, and praxis, and neuroimaging (brain MRI or PET scan) suggesting predominant frontotemporal pathology.^26^ PDB was diagnosed if there was elevated serum alkaline phosphatase (ALP) level and radiographic evidence of mixed lytic and sclerotic lesions resulting in cortical bone thickening, coarse trabeculation, osteoporosis circumscripta in skull, flame‐shaped lesions in long bones, bowed long bones, or increased uptake of radionuclide on bone scan.^27^ Restrictive ventilatory defect was defined as reduced forced vital capacity (FVC) on pulmonary function tests. Maximal inspiratory and expiratory pressures were recorded and considered abnormal if less than 2/3 of predicted. Cardiac function was evaluated by electrocardiogram and transthoracic echocardiogram. A left ventricular ejection fraction of less than 55% indicated systolic dysfunction. Evaluation for left ventricular diastolic function was based on the American Society of Echocardiography and European Association of Cardiovascular Imaging guidelines.^28^ Descriptive data are presented as median and range for continuous variables, and frequency and percent for categorical variables.

## Results

A total of 31 individuals (11 females, 20 males) from 27 unrelated families with genetically proven MSP or MSP‐like disorder were identified. Patients were referred from various areas across United States. Table 1 summarizes genotype and clinical phenotype. Mutations in *VCP* gene were the most common (17 patients/14 families) followed by digenic mutations in *SQSTM1* and *TIA1* (5 patients/5 families) and mutations in *TIA1* (5 patients/4 families). *MATR3*, *HNRNPA1*, *TFG*, and *HSPB8* mutations accounted for single patients. Twenty‐nine patients carried known pathogenic variants. Eleven patients were previously reported.^17^, ^20^, ^29^, ^30^, ^31^ One patient harbored a novel likely pathogenic *VCP* variant, c.1019A > G, p.His340Arg (Family 12/ patient 15). This variant is absent from large control databases, involves a highly conserved amino acid, and is located in the D1 oligomerization domain similarly to other pathogenic *VCP* variants. Its pathogenicity was supported by the myopathological findings of rimmed vacuolar myopathy. One patient carried *SQSTM1* c.1160C > T, p.Pro387Leu (Family 16/ patient 19), which was previously observed in individuals with PDB and FTD but not previously reported in the setting of myopathy. Experimental studies showed that this *SQSTM1* variant impairs its function.^32^, ^33^ The associated distal myopathy with predominant finger extensor weakness, histopathological evidence of myopathy with rimmed vacuoles, and co‐existing *TIA1* c.1070A > G, p.Asn357Ser variant, known to cause myopathy in combination with a *SQSTM1* mutation,^31^, ^34^ supported the pathogenic role of *SQSTM1* c.1160C > T, p.Pro387Leu in the genesis of the myopathy. No patient with *HNRNPA2B1*, *ANXA11, OPTN*, or sole *SQSTM1* (without *TIA1* variant) mutations was identified in the study population. Eighteen (58%) patients had one or more family member with MSP. Two patients from the same family were Hispanic or Latino, one patient was biracial Black and White, the rest were non‐Hispanic White individuals. The median age of symptom onset was 51 years (range 19–75) with limb weakness being the most common initial symptom (n = 28). The 3 remaining patients manifested with dyspnea on exertion (myopathy), fasciculations (myopathy and MND), and cognitive impairment (FTD and MND).

**Table 1:** Genotype and clinical phenotype of patients with multisystem proteinopathy (MSP) and MSP‐like disorders.

Family	Patient	Ancestry	Gene	DNA nucleotide change	Predicted protein change	Phenotypes	Muscle biopsy site
1	1	Italian	*VCP*	c.277C > T	p.Arg93Cys	M (LG), MND, PDB	N/D
2	2	Unknown	*VCP*	c.278G > A	p.Arg93His	M (LG)	Triceps
3	3	German	*VCP*	c.278G > A	p.Arg93His	M (scapulo‐distal), PN	Tibialis anterior
4	4	Irish, Finnish, Portuguese	*VCP*	c.284G > A	p.Arg95His	M (LG), FTD	Supraspinatus
5	5	German, English	*VCP*	c.464G > A	p.Arg155His	M (LG), MND^30^	Biceps brachii
6	6	English, Irish	*VCP*	c.464G > A	p.Arg155His	M (LG)	Tibialis anterior
7	7	German	*VCP*	c.464G > A	p.Arg155His	M (distal), PDB, PN^30^	N/D
	8	German	*VCP*	c.464G > A	p.Arg155His	M (LG)^30^	Deltoid
8	9	Unknown	*VCP*	c.464G > A	p.Arg155His	M (LG), PDB^30^	Gluteus medius
	10	Unknown	*VCP*	n/a^1^	n/a^1^	M (LG), FTD^30^	Biceps brachii
9	11	Mexican	*VCP*	c.476G > A	p.Arg159His	M (LG)	Quadriceps
	12	Mexican	*VCP*	c.476G > A	p.Arg159His	FTD, MND	N/D
10	13	Unknown	*VCP*	c.572G > A	p.Arg191Gln	M (LG)	Quadriceps
11	14	Unknown	*VCP*	c.572G > A	p.Arg191Gln	MND	N/D
12	15	Unknown	*VCP*	c.1019A > G	p.His340Arg	M (LG)	Biceps
13	16	Unknown	*VCP*	c.1156A > G	p.Lys386Glu	M (LG), PDB	Quadriceps
14	17	African‐American	*VCP*	c.1160A > G	p.Asn387Ser	M (scapulo‐distal)^30^	Gluteus medius
15	18	German, Dutch	*HNRNPA1*	c.959A > T	p.Asn320Ile	M (distal)^29^	Tibialis anterior
16	19	Norwegian	*SQSTM1 + TIA1*	c.1160C > T (*SQSTM1*) c.1070A > G (*TIA1*)	p.Pro387Leu p.Asn357Ser	M (scapulo‐distal)	Tibialis anterior
17	20	Irish, Lithuanian	*SQSTM1 + TIA1*	c.1165 + 1G > A (*SQSTM1*) c.1070A > G (*TIA1*)	Splice donor A390X p.Asn357Ser	M (scapulo‐distal)	Deltoid
18	21	Swiss	*SQSTM1 + TIA1*	c.1175C > T (*SQSTM1*) c.1070A > G (*TIA1*)	p.Pro392Leu p.Asn357Ser	M (distal), FTD^31^	Deltoid
19	22	Unknown	*SQSTM1 + TIA1*	c.1175C > T (*SQSTM1*) c.1070A > G (*TIA1*)	p.Pro392Leu p.Asn357Ser	M (scapulo‐distal)^31^	Tibialis anterior
20	23	Norwegian, Danish	*SQSTM1 + TIA1*	c.1273G > A (*SQSTM1*) c.1070A > G (*TIA1*)	p.Gly425Arg p.Asn357Ser	M (distal)	N/D
21	24	Unknown	*MATR3*	c.254C > G	p.Ser85Cys	M (distal)	Tibialis anterior
22	25	Swedish	*TIA1*	c.1150G > A	p.Glu384Lys	M (distal)	Biceps brachii
23	26	Swedish	*TIA1*	c.1150G > A	p.Glu384Lys	M (distal)	N/D
24	27	German	*TIA1*	c.1150G > A	p.Glu384Lys	M (distal), PN	Tibialis anterior
25	28	Swedish	*TIA1*	c.1150G > A	p.Glu384Lys	M (distal)	N/D
	29	Swedish	*TIA1*	c.1150G > A	p.Glu384Lys	M (distal)	Extensor carpi radialis
26	30	Unknown	*TFG*	c.854C > T	p.Pro285Leu	M (scapulo‐distal), MND, PN^17^	Quadriceps
27	31	Norwegian, Portuguese	*HSPB8*	c.577_580dupGTCA	p.T194Serfs*23	M (LG)^20^	Quadriceps

FTD, frontotemporal dementia; LG, limb‐girdle; M, myopathy; MND, motor neuron disorder; N/D, not done; PDB, Paget disease of bone; PN, peripheral neuropathy.

^1^ Genetic data not available as diagnosis were made postmortem. Patient 10 was patient 9's father.

### Myopathy

**Figure 1:**
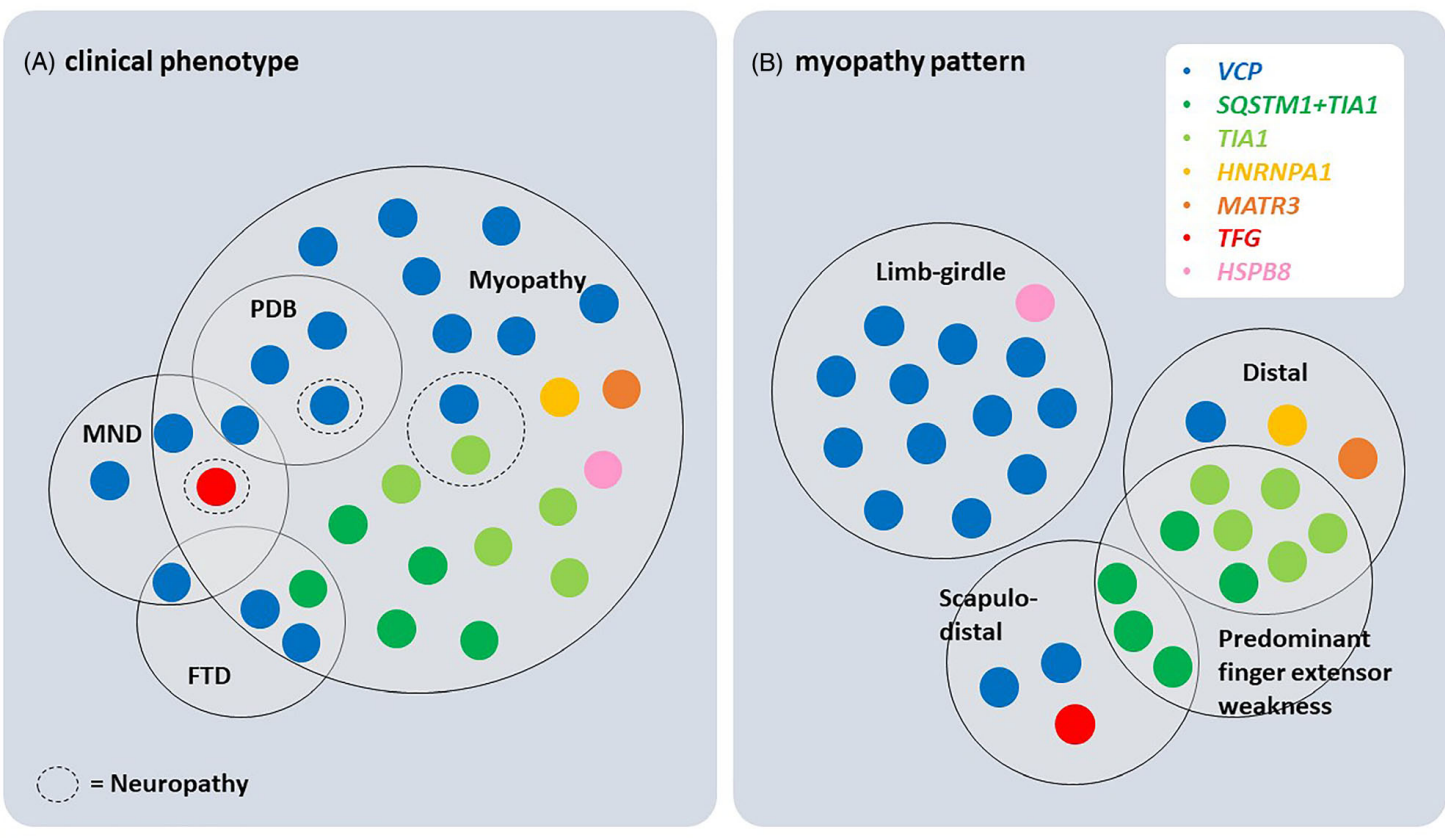
Diagram representing the spectrum of clinical phenotype (A) and myopathy pattern of weakness (B) in MSP and MSP‐like patients described in this study. Each dot represents one patient.

**Figure 2:**
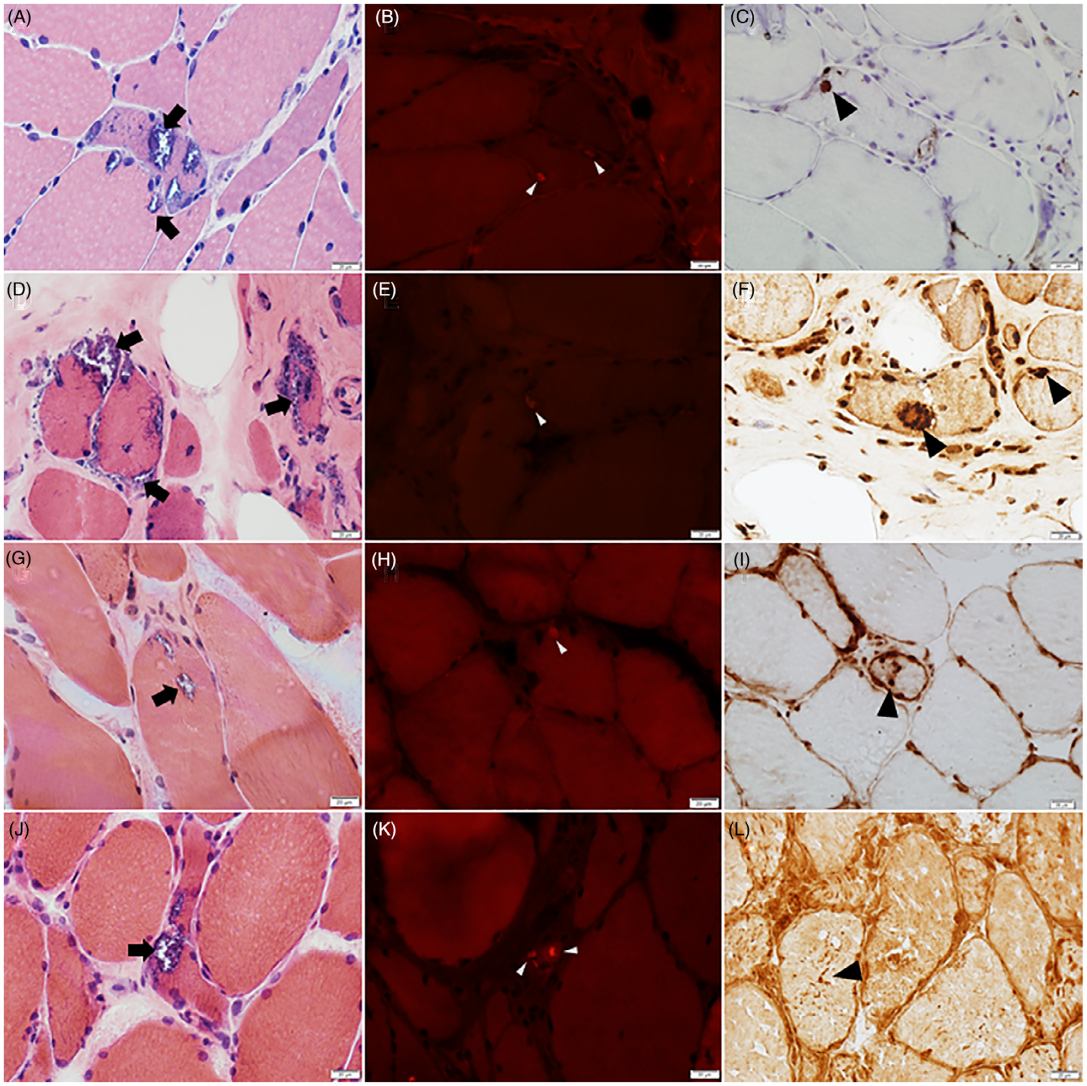
Muscle biopsies from patients with MSP and MSP‐like disorders. Muscle biopsies from patients with *VCP* (A‐C), *HNRNPA1* (D‐F), *SQSTM1 + TIA1* (G‐I), and *HSPB8* (J‐L) mutations. H&E‐stained sections show multiple fibers harboring rimmed vacuoles (arrows, A, D, G, and J). Congo red‐stained sections revealed small sarcoplasmic congophilic inclusions (arrowheads, B, E, H, and K). Immunohistochemical stains demonstrate TDP43‐positive sarcoplasmic inclusions (arrowheads, C and F) and punctate TIA1 sarcoplasmic reactivity (arrowheads, I and L).

Myopathy occurred in all but 2 *VCP*‐MSP patients (*n* = 29, 94%) and was the first or the only clinical manifestation in 27. Median age of myopathy onset was 52 years (range 19–81). The most common pattern of weakness was limb‐girdle (*n* = 13) which occurred only in *VCP* patients and in the *HSPB8* patient (Fig. 1). Distal myopathy occurred in 10 patients (5 *TIA1*, 2 *SQSTM1 + TIA1*, 1 *MATR3*, 1 *HNRNPA*1, 1*VCP*). Predominant weakness of periscapular and distal lower limb muscles with involvement of anterior and posterior compartment (scapulo‐distal) was observed in 6 patients (3 *SQSTM1 + TIA1*,2 *VCP*, 1 *TFG*). All patients with *TIA1* (*n* = 5) and *SQSTM1 + TIA1* (*n* = 5) mutations had predominant finger extensor weakness at presentation. Weakness was asymmetric in 25 (89%) patients. Five *VCP* patients had scapular winging. The patient with *MATR3* mutation and 2 patients with *VCP* mutation had oropharyngeal dysphagia. Only 5 patients reported myalgia (4 *VCP*, 1 *TIA1*). Median CK values were 128 U/L for females and 437 U/L for males (range 77–2,400; normal: < 192, females; < 308, males). CK values above 1,000 U/L were detected in 4 patients (1 *VCP*, 1 *HNRNPA1*, 1 *TIA1*, 1 *HSPB8*). EMG was performed in 28 patients and showed mixed small and large motor unit potentials in 15 (54%), small polyphasic motor unit potentials in 12 (43%), and large motor unit potentials in the patient with *TFG*‐mutation whose phenotype was mainly featured by anterior horn cell disease and sensory neuropathy. Fibrillation potentials with or without myotonic discharges were present in all but one *VCP* patient. Four patients (2 *VCP*, 1 *TFG*, 1 *TIA1*) had also reduced or absent sensory nerve action potentials in upper and lower limbs consistent with axonal sensory neuropathy and length‐dependent sensory deficits on examination. None of them had identified risk factors for the neuropathy. Twenty‐three probands and an affected parent (patient 10) underwent muscle biopsy. The site of muscle biopsy (Table 1) was selected based on clinical examination, targeting a muscle with mild or mild–moderate weakness. Patients with limb‐girdle weakness had biopsy of a proximal muscle; majority of patients with scapulo‐distal weakness underwent biopsy of tibialis anterior while those with distal phenotype had a biopsy of a distal (tibialis anterior, *n* = 3; extensor carpi radialis, *n* = 1) or proximal muscle. Twenty (83%) muscle biopsies showed myopathy with rimmed vacuoles (Fig. 2). The mean number of vacuolated muscle fibers per 20X power field ranged from 0 to 7 (average 2). Most vacuoles were in nonatrophic muscle fibers while about 7% of vacuoles were in atrophic (fiber size <25 μm in diameter) fibers. Congo red‐stained sections viewed under rhodamine optics revealed small sarcoplasmic congophilic inclusions in 15/22 biopsies. Immunohistochemical stains were performed on muscle specimens from 6 patients: Several TDP43‐positive inclusions were present in the muscle biopsy from a *VCP* patient (patient 13); a few p62‐, TDP43‐, and hnRNPA1‐positive sarcoplasmic inclusions were present in the muscle biopsy of the *HNRNPA1* patient^29^; p62‐ and TDP43‐positive sarcoplasmic inclusions were observed in some fibers of a *SQSTM1 + TIA1* patient (patient 20); punctate TIA1 sarcoplasmic reactivity was observed in 2 other *SQSTM1 + TIA1* (patient 21 and 22)^31^ and the *HSPB8* patient.^20^ (Fig. 2) Myofibrillar pathology features were also observed in muscle biopsies of the *HSPB8* patient and two of the *SQSTM1 + TIA1* patient (Patients 19 and 22).^20^, ^31^ Minimal inflammation, consisting of sparse individual inflammatory cells or a single endomysial small inflammatory collection adjacent to structurally abnormal fibers, was present in 11 (48%) biopsies. Nineteen (70%) biopsies showed also pathological features of denervation atrophy with or without reinnervation.

### Motor neuron disorder (MND)

Five (16%) patients (4 *VCP* and 1 *TFG*) had clinical and electrodiagnostic evidence of anterior horn cell disease. Two of the 4 *VCP*‐MSP patients also showed signs of upper motor neuron disease. Three of these 5 patients (2 *VCP* and 1 *TFG*) had concomitant myopathy, two with pathological evidence of myopathy with rimmed vacuoles and one with myopathic EMG features and elevated CK. Median age of onset was 45 years (range 37–57). All 5 patients had weakness that initially and predominantly affected lower limb muscles. The weakness was asymmetric at onset and remained asymmetric at time of clinical evaluation in all patients. In addition, it progressed more slowly compared to classic idiopathic ALS and patients were still able to ambulate with a cane at median of 9 years from weakness onset. Dysphagia occurred in one *VCP* patient. Electrodiagnostic studies showed reduced recruitment of large motor unit potentials with widespread fibrillation and fasciculations potentials in 4 patients. An additional *VCP* patient had fasciculations, hyperreflexia, and Babinski sign but purely myopathic EMG changes apart from the fasciculation potentials.

### Frontotemporal dementia and cognitive impairment

Cognitive impairment was identified in 4 (13%) patients (3 *VCP* and 1 *SQSTM1 + TIA1*) through short cognitive evaluation and formal neuropsychological testing. Median age of onset of cognitive symptoms was 56 years (range 51–72) among the 3 *VCP* patients and 73 years in the *SQSTM1 + TIA1* patient. One *VCP* patient had cognitive impairment as initial manifestation. The other three patients developed cognitive and behavioral symptoms 13, 14, and 16 years after the myopathic symptoms, respectively. Impairments were predominantly in the frontotemporal domains, with compromised verbal fluency (*n* = 4), executive dysfunction (*n* = 3), apathy (*n* = 2), or perseverative/compulsive behavior (*n* = 1). Visuospatial skills were mildly impaired in 2 patients. MRI brain, performed in 3 of 4 patients with cognitive symptoms, showed frontal and anterior temporal lobe volume loss in one patient and mild generalized volume loss with confluent periventricular and deep white matter hyperintensities in the others. Two patients (1 *VCP* and 1 *SQSTM1 + TIA1*) underwent brain PET‐CT which demonstrated frontotemporal hypometabolism in both patients. One patient who was evaluated in 1980 s did not have brain imaging. Seven patients (5 *VCP*, 1 *SQSTM1 + TIA1*, 1 *TFG*) without symptoms or signs of cognitive impairment underwent brain MRI 8 years (median, range 2–20) after the onset of the weakness: 1 (*VCP*) demonstrated mild atrophy of the medial anterior temporal lobes, 2 (*VCP*) showed mild generalized atrophy, and the remaining 4 had normal findings.

### Paget disease of bone

PDB was identified in 4 (13%) patients, all with *VCP* mutations, by elevated ALP and radiological studies. Median age of detection was 57 years (range 47–60). The diagnosis of PDB was made 6–13 years after the myopathy onset. One patient presented with bone pain and unilateral hearing loss; the others were asymptomatic. Median ALP, in the patients diagnosed with PDB, was 143.5 IU/L (range 112–336; normal <129). The bones involved were pelvic (*n* = 3), spine (*n* = 1), and proximal femur (*n* = 1). Two patients received zolendronic acid therapy with improvement of bone pain (in the symptomatic patient) and ALP levels. The other two patients were asymptomatic, had minimally elevated ALP, which remained stable during the follow‐up period, and did not require pharmacotherapy. Three additional *VCP*‐MSP patients had mildly elevated ALP (144–159 IU/L) without evidence of PDB by bone radiographs or radionucleotide scans. In addition, screening bone scan or bone survey were performed in 6 asymptomatic patients with normal ALP (4 *VCP*, 2 *SQSTM1 + TIA1*) and were normal.

### Respiratory and cardiac findings

Twelve patients (9 *VCP*, 2 *SQSTM1 + TIA1*, 1 *TFG*) had pulmonary function tests at median of 15 years (range 5–22) after disease onset: 6 showed restrictive ventilatory defect (FVC 70–80% of predicted in 3 patients, FVC <70% of predicted in 3 patients) with median vital capacity of 2.1 L (range 1.8–3.8); the other 6 had reduced maximum inspiratory or expiratory pressure suggestive of neuromuscular weakness. Restrictive ventilatory defect was present in 4/26 (15%) patients with myopathy compared to 2/5 (40%) patients with MND at the time of testing. One patient (*TFG*) required nocturnal bilevel positive airway pressure 8 years after the onset of weakness with improvement of daytime hypersomnolence.

Electrocardiogram was performed in 25 patients and demonstrated baseline sinus rhythm in all. Twenty‐one patients had one or more echocardiogram (at median of 8 years from initial symptom onset, range 3–22 years). No patient had left ventricular systolic dysfunction; median ejection fraction was 64%. Diastolic dysfunction was noted in 2 *VCP* patients. Increased concentric left ventricular wall thickness was observed in 3 *VCP* and 1 *TIA1* patients. This latter patient, however, had also a pathogenic *MYH7* variant (c.5459G > A, p.Arg1820Gln) contributing to the phenotype.^35^ No patients required a pacemaker or a defibrillator.

### Long‐term follow‐up: disease progression and complications

Median follow‐up time was 11.5 years (range 2–26) from symptom onset. Median age at last follow‐up was 64 years (range 25–84). Figure 1 summarizes patient's phenotype at last follow‐up visit: 18 (58%) patients had isolated myopathy, 3 (10%) myopathy with FTD, 3 (10%) myopathy with PDB, 2 (6%) myopathy with MND, 1 (3%) myopathy with MND and PDB, 1 (3%) MND with FTD, and 1 (3%) MND. Four patients had associated axonal neuropathy. MND, myopathy with rimmed vacuoles, and sensory neuropathy were all present at time of diagnosis in the patient with *TFG* mutation. Isolated myopathy at time of last follow‐up was much less frequent in *VCP* patients (41%) compared to patients with mutations in other genes (79%), *p* = 0.04. No patient developed parkinsonism during follow‐up. All patients were evaluated by physiatrists who provided individualized recommendations regarding an exercise program, physical and occupational therapy, and devices to improve functionality based on specific needs. Fifteen (48%) patients were able to ambulate independently without gait‐aid at last follow‐up. Thirteen (42%) patients (with myopathy, *n* = 9; MND, *n* = 1; myopathy and MND, *n* = 3) started using a cane or a walker at median of 10 years (range 3–21) after weakness onset. Two *VCP* patients with myopathy had a more rapid progression of weakness and required full‐time wheelchair 6 and 9 years after symptom onset, respectively. These 2 patients did not have clinical features of MND, but an EMG study was not repeated after they became nonambulatory. Therefore, the possibility that they may have developed MND cannot be entirely excluded. Six out of 16 patients who presented with distal or scapulo‐distal phenotype required ankle‐foot orthoses at median of 5 years (range 1–20) after the myopathy onset. Three *VCP*‐MSP patients died 18–24 years after disease onset, 2 at the age of 66 and 1 at the age of 59. The cause of death was respiratory infection in one patient and unknown in the other two. Of note, one *VCP* patient developed colorectal cancer during the time of follow‐up (at age 66) and 3 other patients (1 *VCP*, 2 *SQSTM1 + TIA1*) had prostate cancer.

### Clinical comparison among classic MSPs and MSP‐like disorders

We clustered the patients in 3 groups based on genotype to compare their clinical characteristics: 1. *VCP*, 2. Other *classic* MSP genes (*HNRNPA2B1, HNRNPA1*, and *SQSTM1* + *TIA1*), 3. MSP‐like disorder genes (*TIA1*, *MATR3*, *TFG*, and *HSPB8*) (Table 2). There were no significant differences in frequency of clinical features across genotype, although PDB occurred exclusively in *VCP*‐MSP in our cohort. Myopathy with rimmed vacuoles was the most common clinical phenotypes among all groups with no difference in estimated vacuolar frequency among genotypes. However, patients with *VCP*‐MSP presented more frequently with limb‐girdle weakness compared to patients with non‐*VCP* MSP (*p* = 0.002) and MSP‐like disorders (p = 0.006). The age of onset was older in non‐*VCP* MSP patients: 6^th^ decade in non‐*VCP* MSP versus 5^th^ decade in the other two groups. Such difference, however, could be due to the fact that 5 of the 6 patients in the non‐*VCP* MSP group had the *SQSTM1+* *TIA1* mutations associated with late‐onset myopathy and therefore skewing the results.

At long‐term follow‐up, left ventricular diastolic dysfunction, loss of ambulation, and death were only observed in the *VCP*‐MSP group.

**Table 2:** Comparison of clinical characteristics between patients with *VCP* mutations versus patients with mutations in other MSP‐causative genes (*HNRNPA2B1, HNRNPA1*, and *SQSTM1*) and patients with mutations in genes associated with MSP‐like disorders (*TIA1*, *MATR3*, *TFG*, and *HSPB8*).

Clinical characteristics	*VCP*‐MSP (*n* = 17)	non‐*VCP* MSP (*n* = 6)	*p*‐value^1^	MSP‐like disorder (*n* = 8)	*p‐*value^2^
Female sex, *n* (%)	7 (41%)	1 (17%)	0.37	3 (38%)	1.00
Age at symptom onset, years, median (range)	49 (40–63)	63(52–81)	0.003*	50 (19–58)	0.73
Family history of MSP, *n* (%)	12 (71%)	2 (33%)	0.16	4 (50%)	0.39
Clinical phenotypes, *n* (%)					
Myopathy	15 (88%)	6 (100%)	1.00	8 (100%)	1.00
Motor neuron disorder	4 (24%)	0	0.54	1 (13%)^4^	1.00
Frontotemporal dementia	3 (18%)	1 (17%)^3^	1.00	0	0.53
Paget disease of bone	4 (24%)	0	0.54	0	0.27
Neuropathy	2 (12%)	0	1.00	2 (25%)	0.57
Limb‐girdle pattern of weakness, *n* (%)	12/15 (80%)	0	0.002*	1 (13%)^5^	0.006*
Histopathological evidence of myopathy with rimmed vacuoles, *n* (%)	10/13 (77%)	4/5 (80%)	1.00	6/6 (100%)	0.52
Number of muscle fibers with rimmed vacuoles per 20X power field, mean (range)	2 (0–4)	4 (0–7)	0.22	1 (0–3)	1.00
Left ventricular diastolic dysfunction, *n* (%)	2/13 (15%)	0/3	1.00	0/4	1.00
Restrictive ventilatory defect, *n* (%)	4/9 (44%)	1/1^3^	1.00	1/1^4^	1.00
Age at last follow‐up, years, median (range)	60 (50–84)	74 (62–83)	0.01*	63 (25–74)	0.95
Disease duration at last follow‐up, years, median (range)	12 (3–25)	8 (2–15)	0.07	12 (6–26)	0.73
Isolated myopathy at last follow‐up, *n* (%)	8 (47%)	5 (83%)	0.18	7 (88%)	0.09
Loss of ambulation at last follow‐up, *n* (%)	5 (29%)	0	0.27	0	0.14
Death during time of follow‐up, *n* (%)	3 (18%)	0	0.54	0	0.53
Age at death, years, median (range)	66 (59–66)				

^1^ *p*‐value: *VCP*‐MSP versus non‐*VCP* MSP.

^2^ *p*‐value: *VCP*‐MSP versus MSP‐like disorder.

^3^ This patient has *SQSTM1 + TIA1* mutations.

^4^ This patient has *TFG* mutation.

^5^ This patient has *HSPB8* mutation.

* Statistically significant.

## Discussion

The genetic defects underlying our patients' MSP and MSP‐like disorder were heterogeneous. Despite the growing list of genes associated with such disorders, mutations in *VCP* were the most common and accounted for about half of all MSPs, similar to prior report.^36^ Mutations in *TIA1* and digenic mutations in *SQSTM1* + *TIA1* followed, each occurring in about 1/6 of patients in our cohort. Disease‐causing mutations in *HNRNPA1, MATR3, HSPB8*, and *TFG* accounted for single cases, in keeping with their rarity.^37^, ^38^ We did not find any patients with *HNRNPA2B1* mutations which are also very rare.^37^, ^38^ No patient carrying *ANXA11* mutation was identified, but this could be due to the recent discover of *ANXA11* (not included in NGS panels targeting genes causative of neuromuscular disorders or ALS/FTD at the time of patient's evaluation) as MSP‐causative gene,^14^ and retrospective nature of the study.

Myopathy with rimmed vacuole was the most common phenotype (about 90% of patients) in our cohort and usually the first manifestation of all MSPs and MSP‐like disorders, and not limited to *VCP*‐MSP.^8^ Although MSPs are known to affect multiple organ systems as the disease progresses, myopathy was the only clinical manifestation in most patients with various genotype (79%) even after 10 years from disease onset. Motor neuron disorder occurred in 16% of patients. FTD and PDB were each diagnosed in 13% of patients. The combination of all three main MSP features (myopathy, ALS/FTD, PDB) was not observed in our cohort, consistent with the previously reported rarity of MSP patients with a complete triad.^3^, ^8^ Because most MSP‐ and MSP‐like patients presents with myopathy, muscle biopsy can play an important role in achieving the diagnosis, especially when family history is lacking. Based on our results, the large majority of MSP and MSP‐like muscle biopsies showed myopathy with rimmed vacuoles, including 77% of *VCP*‐MSP, which is much more than previously reported (40%) in prior *VCP*‐MSP cohort.^8^ Such difference in vacuolar change frequency could be related to the biopsy anatomical site. This, at some centers, may be limited to easily accessible muscles, which could be only marginally affected at time of the procedure. Additionally, vacuole distribution can be patchy within a muscle, as suggested by our estimated number of vacuolated muscle fibers ranging from 0 to 7 per 20X power field. Thus, the size of muscle biopsy specimen would also affect the yield of vacuole detection. No difference in rimmed vacuoles frequency was observed among patients with MSP and MSP‐like disorders who had a muscle biopsy.

Although isolated MND has been reported in patients with mutations in various MSP‐ and MSP‐like genes (*VCP, HNRNPA1*, *HNRNPA2B1 SQSTM1*, and *MATR3*)^3^, ^10^, ^29^, ^39^, ^40^, ^41^ MND often coexisted with myopathy in our cohort. Co‐existence of unexplained upper or lower motor neuron involvement or electrophysiological features of lower motor neuron disease in patients with myopathy should raise suspicion for MSP and MSP‐like disorders. Thirteen percent of patients manifested cognitive impairment (*VCP and SQSTM1* + *TIA1*). FTD age of onset among *VCP*‐MSP patients was 56 years, in keeping with prior reports.^8^, ^39^ Patients and families should be educated about the risk of developing FTD, when carrying mutations in genes known to cause also FTD. In our cohort, genetic counseling was provided to all patients and their families. Moreover, genetic diagnosis in the proband led to targeted mutational analysis and diagnosis in several affected family members.

PDB occurred infrequently, affecting only 24% of *VCP*‐MSP. Although most patients had alkaline phosphatase measured, not all underwent radiological studies to search for PDB; query about PDB‐related symptoms may have been limited. Detection of PDB is relevant as it is pharmacologically treatable.^31^ Our results suggest that *VCP*‐MSP patients benefit from bisphosphonates in reducing bone pain and alkaline phosphatase level. Based on current literature, screening for PDB should be performed in patients with *VCP, HNRNPA2B1, HNRNPA1*, and *SQSTM1* mutations, but considering the defective molecular pathways underlying other MSP and MSP‐like disorders, it would not be surprising detecting bone involvement in all these disorders.

No significant genotype–phenotype correlation has been identified in MSP and MSP‐like disorders with the exception of *TIA1* c.1150G > A (p.Glu384Lys) resulting in isolated Welander distal myopathy,^12^ as observed also in our cohort. For *VCP*, *HNRNPA2B1*, *HNRNPA1*, *SQSTM1*, or *MATR3*, both inter‐ and intrafamilial phenotype variability has been observed.^3^, ^9^, ^10^, ^29^, ^39^, ^42^ Reports on small number of cases have linked specific *VCP* mutations exclusively to Charcot–Marie‐Tooth^43^ or dementia.^44^ A large multicenter study associated *VCP* c.463C > T (p.Arg155Cys) with earlier disease onset (30 s‐40 s), higher frequency of axial‐upper limb weakness, and cognitive impairment.^45^ Recently, *HNRNPA2B1* heterozygous frameshift variants were discovered in early‐onset myopathy with ophthalmoparesis without cognitive or bone involvement, but the oldest patient was in his early 40s.^46^ The understanding of these disorders phenotypic variability is limited. Genetic modifiers likely play a role, such as the synergistic effect of *TIA1* (c.1070A > G, p.Asn357Ser) and *SQSTM1* mutations affecting the UBA domain, resulting in distal myopathy.^31^, ^34^ Limited investigations in patients with a specific predominant phenotype (e.g., difficulty assessing muscle strength reliably in patients with FTD, little tendency to obtain formal neuropsychological assessment in IBM patients, lack of myopathological correlate in patients diagnosed with peripheral neuropathy) and the limited information on natural history of the disease, especially for non‐*VCP*‐MSPs, could also contribute to the reported phenotypic variability. It is possible that our current knowledge on these disorders may only represent the tip of the iceberg and convergence of myopathy, MND, FTD, and PDB might occur more frequently than currently known.

All patients evaluated by pulmonary function tests 15 years after disease onset demonstrated respiratory muscle involvement while only 2 of the 17 *VCP* patients had low‐grade left ventricular diastolic dysfunction. Such data, as well as published data,^45^, ^47^, ^48^ suggest the need for monitoring pulmonary and cardiac functions in patients with MSP and MSP‐like disorders. The progression of limb weakness, either from myopathy or MND, was relatively slow with most IBM patients (84%) still ambulating 10 years after symptom onset, more than half without a cane or walker. Loss of ambulation exclusively occurred in *VCP*‐MSP patients, 29% at a median of 12 years follow‐up, in keeping with the recently reported data of 23% at 8.5 years.^45^ Supportive care, including physical and occupational therapy, and prompt access to gait aids to improve functionality, was crucial. Death occurred 18–24 years after VCP disease onset and typically in the 6^th^ decade, like previously reported.^39^ Rare tumors, recently reported in *VCP*‐MSP, were not observed in our patients.^49^

Limitations of this study are inherent to its retrospective design and sample size. Data were not homogeneously collected; non‐neurological complications may have been undetected as pertinent diagnostic studies were not performed, and mortality rate may have been underestimated. Additional limitations include the lack of patient's assessment by functional and quality of life scales, which would have provided a more complete view of the patient's functional state and disease progression. Moreover, because our search targeted patients with genetically proven diagnosis, their number may have been underestimated. Thus, some patients with myopathological features of IBM without identified pathogenic mutations by NGS or patients with ALS who did not undergo genetic testing may or may not have MSP or MSP‐like disorder.

In conclusion, clinicians should be aware that MSP and MSP‐like disorders often manifest without the full spectrum of MSP and that myopathy with rimmed vacuoles is the most common initial presentation. Such pathological phenotype should trigger a search for other tissue involvement as well as a definite genetic diagnosis. This approach will allow clinicians to provide reliable genetic counseling as patients with myopathy with rimmed vacuoles due to MSP or MSP‐like causative genes and their family members may be at risk of developing a more devastating diseases, such as motor neuron disorder or FTD.

## Author Contributions

Pitcha Chompoopong and Margherita Milone involved in conception and design of the study, acquisition, and analysis of the data, drafting the manuscript. Björn Oskarsson, Nicolas N. Madigan, Igal Mirman, Jennifer M. Martinez‐Thompson, and Teerin Liewluck involved in acquisition of the data and revision of the manuscript for content.

## Conflict of Interest

MM has received honoraria as associate editor of Neurology Genetics, and for serving in the Argenx advisory board for a topic unrelated to this project.

## Funding

Study funded by Regenerative Medicine Minnesota (P008848103) to M.M.
